# The Effects of Dietary Fat and Iron Interaction on Brain Regional Iron Contents and Stereotypical Behaviors in Male C57BL/6J Mice

**DOI:** 10.3389/fnut.2016.00020

**Published:** 2016-07-21

**Authors:** Lumei Liu, Aria Byrd, Justin Plummer, Keith M. Erikson, Scott H. Harrison, Jian Han

**Affiliations:** ^1^Department of Biology, North Carolina Agricultural and Technical State University, Greensboro, NC, USA; ^2^Department of Nutrition, The University of North Carolina at Greensboro, Greensboro, NC, USA

**Keywords:** behavior, brain, iron, high-fat diet, ferritin-H

## Abstract

Adequate brain iron levels are essential for enzyme activities, myelination, and neurotransmitter synthesis in the brain. Although systemic iron deficiency has been found in genetically or dietary-induced obese subjects, the effects of obesity-associated iron dysregulation in brain regions have not been examined. The objective of this study was to examine the effect of dietary fat and iron interaction on brain regional iron contents and regional-associated behavior patterns in a mouse model. Thirty C57BL/6J male weanling mice were randomly assigned to six dietary treatment groups (*n* = 5) with varying fat (control/high) and iron (control/high/low) contents. The stereotypical behaviors were measured during the 24th week. Blood, liver, and brain tissues were collected at the end of the 24th week. Brains were dissected into the hippocampus, midbrain, striatum, and thalamus regions. Iron contents and ferritin heavy chain (FtH) protein and mRNA expressions in these regions were measured. Correlations between stereotypical behaviors and brain regional iron contents were analyzed at the 5% significance level. Results showed that high-fat diet altered the stereotypical behaviors such as inactivity and total distance traveled (*P* < 0.05). The high-fat diet altered brain iron contents and FtH protein and mRNA expressions in a regional-specific manner: (1) high-fat diet significantly decreased the brain iron content in the striatum (*P* < 0.05), but not other regions, and (2) thalamus has a more distinct change in FtH mRNA expression compared with other regions. Furthermore, high-fat diet resulted in a significant decreased total distance traveled and a significant correlation between iron content and sleeping in midbrain (*P* < 0.05). Dietary iron also decreased brain iron content and FtH protein expression in a regionally specific manner. The effect of interaction between dietary fat and iron was observed in brain iron content and behaviors. All these findings will lay foundations to further explore the links among obesity, behaviors, and brain iron alteration.

## Introduction

Two-thirds of the population in the US are considered overweight or obese, and this number continues to rise. Obesity-associated diseases, including neurological disorders, diabetes, and cardiovascular illness, impose an enormous burden ($150 billion annually) on the US public health system. One of the most recently studied obesity-associated disorders, systemic iron deficiency (ID), has sparked attention. National and international epidemiological data have shown ID to be two times more likely in overweight and obese children than in the children of normal weight (1–5).

The effect of obesity on iron metabolism has been studied in both human and animal models. An inverse relationship between body mass index (BMI) and plasma iron concentrations was observed in obese children (1). Genetic obese adult mice (ob/ob) showed lower iron concentrations in liver, muscle, femur, bone, and plasma than lean mice (6, 7). Dietary-induced obese mice showed a significant decrease of hepatic non-heme iron contents after 16 weeks of high-fat treatment (8). Furthermore, a decreased liver iron content and increased inflammation (hepcidin and IL-6) in adipose tissue was observed in a 24-week dietary-induced Swiss mice model (9). All above studies focused on body iron stores or status affected by obesity. However, the brain regional iron content affected by high-fat diet has not yet been explored.

Brain iron is essential for biological processes such as oxygen delivery, myelination, and neurotransmitters synthesis (10, 11). Brain iron disturbance leads to molecular, metabolic, structural, and synaptic changes that resulted directly in behavioral outcomes as demonstrated in human and rodent studies (12–14). In humans, early childhood ID results in poor inhibitory control, learning obstacles, poor cognition, and motor performance (15–18). ID during infancy leads to negative impact on executive function and memory (19). In rodents, brain iron disturbance was associated with increased anxiety and poor performance on memory tasks (20–24). Therefore, investigation of brain iron dysregulation under obesity or other disease conditions are very important.

The brain regions known to be sensitive to iron status changes are the hippocampus, striatum, midbrain, and thalamus (25–30). These iron-rich regions control various behaviors. For examples, the hippocampus handles spatial memory, navigation, and olfaction (31). The striatum plans and modulates movement pathways (32) and facilitates and balances stimuli (33). The midbrain controls multiple functions including circadian system, vision, hearing, motor control, arousal (alertness), and temperature regulation (34, 35). The substantia nigra (SN) is the major component of the midbrain. SN controls eye movement, motor planning, reward seeking, and learning. The death of dopaminergic neurons in the midbrain results in Parkinson’s disease and associated abnormal behaviors including tremor, bradykinesia, stiffness, disturbances to posture, fatigue, sleep abnormalities, and depressed mood (36, 37). The thalamus is the subcortical center of the motor control network (38). It regulates relaying sensory and motor signals to the cerebral cortex, regulating consciousness, sleep, wakefulness, and alertness (39, 40).

The changes in iron contents in the hippocampus, midbrain, striatum, and thalamus have been widely studied under the conditions of ID. However, under the fast growing epidemics of obesity, the effect of obesity on regional iron changes has not been examined. The objective of this study was to investigate the impacts of dietary fat and iron interaction on brain regional iron contents and associated behavior patterns. The results will provide novel information to obesity research and its relation to nutrition and brain neurology.

## Materials and Methods

### Animal Subjects

Thirty four-week-old C57BL/6J male mice (Jackson Laboratories, Bar Harbor, ME, USA) were randomly assigned to six dietary treatment groups (*n* = 5). These diets were 10% kcal derived from fat + 36.9 mg Fe/kg diet (control fat control iron, CF/CI, Cat# D13010403), 10% kcal derived from fat + 529 mg Fe/kg diet (control fat high iron, CF/HI, Cat# D13010405), 10% kcal derived from fat + 3.6 mg Fe/kg diet (control fat low iron, CF/LI, Cat# D13010401), 45% kcal derived from fat + 36.9 mg Fe/kg diet (high fat control iron, HF/CI, Cat# D13010404), 45% kcal derived from fat + 529 mg Fe/kg diet (high fat high iron, HF/HI, Cat# D13010406), and 45% kcal derived from fat + 3.6 mg Fe/kg diet (high fat low iron, HF/LI, Cat# D05101905) (Research Diets Inc., New Brunswick, NJ, USA). Other major ingredients in the experimental diets, expressed as grams per kilogram diet, include casein (200 g/kg), l-Cystine (3 g/kg), cornstarch (high fat: 452.2 g/kg, low fat: 72.8 g/kg), sucrose (172.8 g/kg), cellulose (50 g/kg), soybean oil (25 g/kg), mineral mix S18708 (10 g/kg), and vitamin mix V10001 (10 g/kg). Diets and de-ionized water were given to mice *ad libitum*. Mice were housed individually in a temperature-controlled room. The room temperature was maintained at 25 ± 1°C, with each dark cycle occurring between 7 p.m. and 7 a.m. daily.

Dietary intake and mice body weight were measured weekly. During the 24th week, mice from each treatment group (*n* = 5), except mice fed with HF/CI diet, were recorded for stereotypical behaviors. Mice fed with HF/LI diet developed ulcerative dermatitis and were euthanized at the end of 16th week after stereotypical behaviors were recorded. The rest of the treatment groups were euthanized at the end of the 24th week_._ Blood, liver, and brain tissues were collected. Brains were dissected into the hippocampus, midbrain, striatum, and thalamus. Tissues were snap frozen in liquid nitrogen and stored at −80°C. Hematocrit levels were measured using a micro-capillary centrifuge (Model MB, IEC, Needham Heights, MA, USA) by spinning samples at 10,000 rpm for 10 min. Hemoglobin concentrations were measured at 540 nm following the manufacture’s protocol (Sigma #9008-020).

All studies were conducted in an American Association for Laboratory Animal Care-accredited facility following protocols approved by the Institution of Animal Care and Use Committee (IACUC) at the University of North Carolina at Greensboro (UNCG). The procedures were performed by the principles and guidelines established by the National Institutes of Health for the care and use of laboratory animals.

### Liver Triglyceride Extraction and Measurement

Liver triglyceride (TG) concentrations were determined by a colorimetric assay (41). Briefly, liver tissues (100–300 mg) were weighed and placed into ethanolic potassium hydroxide solution (1 part of 100% ethanol:2 parts of 30% KOH). The mixture was incubated at 55°C overnight and then centrifuged at 14,000 rpm at 4°C for 5 min. The supernatant was mixed with 1M magnesium chloride (MgCl_2_) and incubated on ice for 10 min. The mixture was centrifuged at 14,000 rpm at 4°C for 5 min. The supernatant was taken to measure TG content using free glycerol reagent (Cat#F6428, Sigma-Aldrich, St Louis, MO, USA) and glycerol standards (Cat#G7793, Sigma-Aldrich, St Louis, MO, USA) following the manufacturer’s protocol.

### Brain Iron Content Measurement

Brain samples were sonicated in the cold radioimmunoprecipitation assay buffer (1% non-idet-P40, 0.1% sodium dodecyl sulfate, 0.5% sodium deoxycholate in the phosphate saline buffer, pH = 7.5) containing protease inhibitors. Separate aliquots of each sonicated sample were assayed for protein concentration [Pierce Bicinchoninic Acid (BCA) Protein Assay Thermo Fisher Scientific Inc., Rockford, IL, USA] and iron content. Samples used for the iron assay were digested in ultrapure nitric acid (1:10 ratio) for 48 h in a sand bath (60°C). Aliquots (20 μl) of digested homogenate were further diluted with 2% nitric acid for analysis. Iron concentrations were measured using graphite furnace atomic absorption spectrometry (Varian AA240, Varian, Inc., USA). Bovine liver (NBS Standard Reference Material, USDC, Washington, DC, USA) containing 184 μg Fe/g was digested in ultrapure nitric acid and used as an internal standard for analysis. All samples and controls were run in triplicates. Iron contents were expressed as micrograms of iron per milligram of protein.

### Western Blot to Detect the Protein Expression of Iron-Related Proteins

Brain and liver samples from each treatment were homogenized in RIPA buffer with protease inhibitor in a 1:10 weight:volume ratio. Samples were spun at 15,000 and 14,000 rpm, respectively. Aliquots of the homogenates were used to determine protein concentration using the BCA Protein Assay. Equal amounts of lysate proteins were separated by sodium dodecyl sulfate polyacrylamide gel electrophoresis (SDS-PAGE), transferred onto nitrocellulose membranes, and immunoblotted with primary antibodies. Ferritin heavy chain (FtH) and light chain (FtL) primary antibodies (abcam) were diluted in 1:500 to determine the brain or liver protein expression. Glyceraldehyde 3-phosphate dehydrogenase (GAPDH) (Novus Biological) and β-Actin (abcam) were all diluted in 1:1000. Mouse monoclonal or polyclonal secondary antibodies (Bio-Rad laboratories) were used in 1:3000 dilutions. The protein bands were visualized using SuperSignal West Pico chemiluminescent substrate Western blotting detection reagents (thermo scientific) and X-ray film (GeneMate).

### Real-time PCR to Detect the FtH mRNA Expression

Real-time PCR gene expression assay was conducted for FtH gene (Mm00850707_g1). Twenty nanograms of FtH cDNA were used in the TaqMan^®^ Universal Master Mix II (Cat#4440040, Life Technologies, Carlsbad, CA, USA), and the assay was performed according to the manufacturer’s protocol. Control and target assays were validated on excess sample tissue (*n* = 5). The 18s gene assay (Hs99999901_s1) was selected as the appropriate endogenous control. Relative gene expression was quantified using the delta delta *C*_t_ method.

### Behavioral Analysis

Behavioral analysis was conducted using the Clever Home Cage Scan (HCS) system (Clever Systems Inc., Reston, VA, USA) (42, 43). The HCS system utilized video images from the home cage acquired at 30 frames/s. Based on the sequential postures of the mice and position of body parts in space, behaviors were assigned using pre-trained data sets as a reference. The computer software categorized these behaviors into different categories (44). The agreement between behaviors identified by the HCS and mice actual behavior was ≥90% (42). During 24 weeks of the dietary treatments, each mouse was placed in an individual shoebox cage with food, water, and minimal bedding. Before recording, mice were acclimated for 24 h in the recording room to ensure that any behavior alterations captured were treatment effects. After acclimation, mouse activities, such as total distance traveled (TDT), inactivity, sleeping, grooming, sniffing, foraging, feeding, and twitching, were recorded by video surveillance for 24 h. These data were exported to Microsoft Excel 2007, Prism 5, and SPSS for graphs and statistical analysis.

### Statistical Analyses

Multivariate analysis (two-way ANOVA) was used to analyze the effect of dietary fat and iron, respectively, as well as the interaction effect of dietary iron and fat, on all data outcomes (*P* < 0.05). When there is no interaction effect of dietary iron and fat, one-way ANOVA was used to test the significant effect of high-fat diet at all iron levels of all data outcomes (*P* < 0.05). The *Post Hoc* analysis was used to test the effect of dietary iron at both fat levels on all data outcomes (*P* < 0.05). When there is an interaction effect of dietary iron and fat, an independent *t*-test was used to compare the differences between control fat group and high-fat group at the same iron level of all data outcome (*P* < 0.05). All statistical analyses were done in SPSS.

## Results

### Physiological Data

**Figure 1:**
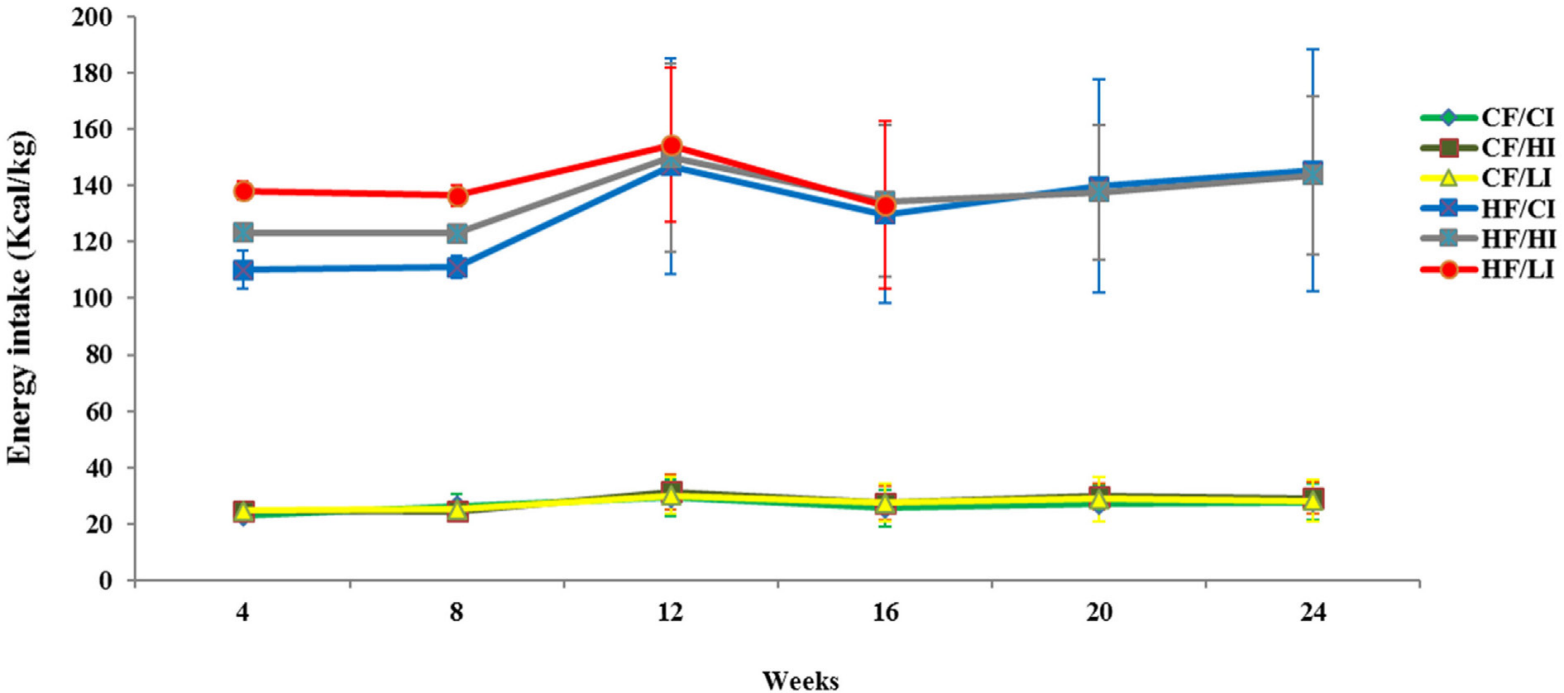
**Weekly energy intake per mouse from initial week to terminal week**. Abbreviations: CF, control fat; HF, high fat; CI, control iron; HI, high iron; LI, low iron.

The average dietary intake per mice per week was converted to energy intake shown in Figure 1. Dietary fat and iron, respectively, had a significant effect on the energy intake (*P* < 0.05). Mice fed with high fat diet had significantly higher energy intake than mice fed with the control fat diet at all iron levels (HF vs. CF, *P* < 0.05).

**Figure 2:**
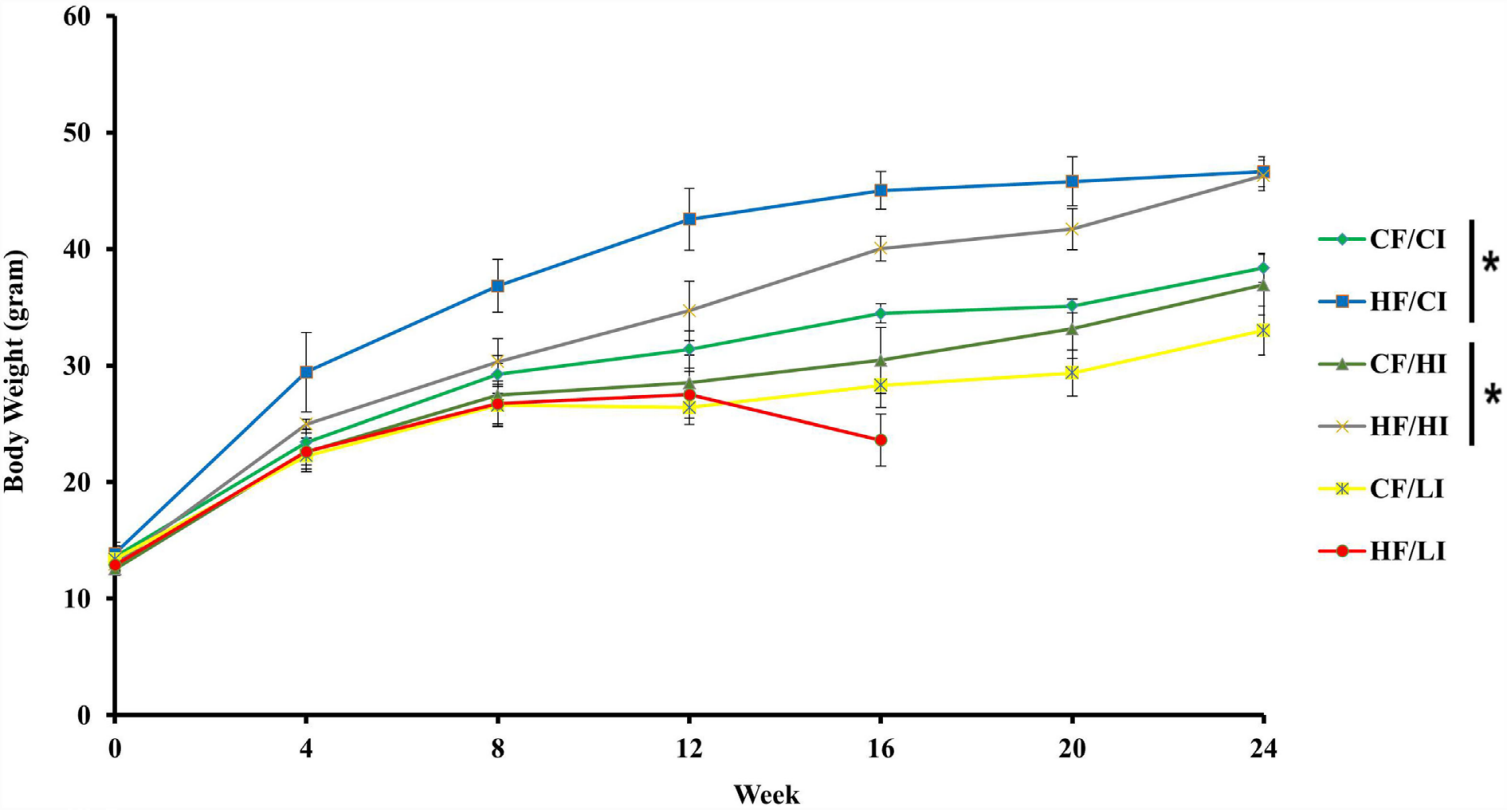
**Mice average body weight per treatment group from the initial week to terminal week**. * represents a significant difference in average dietary intake between control and high fat-treated groups (one-way ANOVA, *P* < 0.05). Abbreviations: CF, control fat; HF, high fat; CI, control iron; HI, high iron; LI, low iron.

The effects of dietary fat and iron on body weight were shown in Figure 2. Dietary fat and iron, respectively, had a significant effect on body weight (*P* < 0.05). There was an interaction between dietary fat and iron on body weight (*P* < 0.05). Mice fed with high fat diets, at both control and high iron levels, had a significant higher body weight compared with their control fat fed pairs (HF/CI vs. CF/CI, HF/HI vs. CF/HI, *P* < 0.05). Mice fed with low iron diet, at both control and high fat levels, had a decreased body weight compared with control iron pairs (CF/CI vs. CF/LI, HF/CI vs. HF/LI, *P* < 0.05).

**Figure 3:**
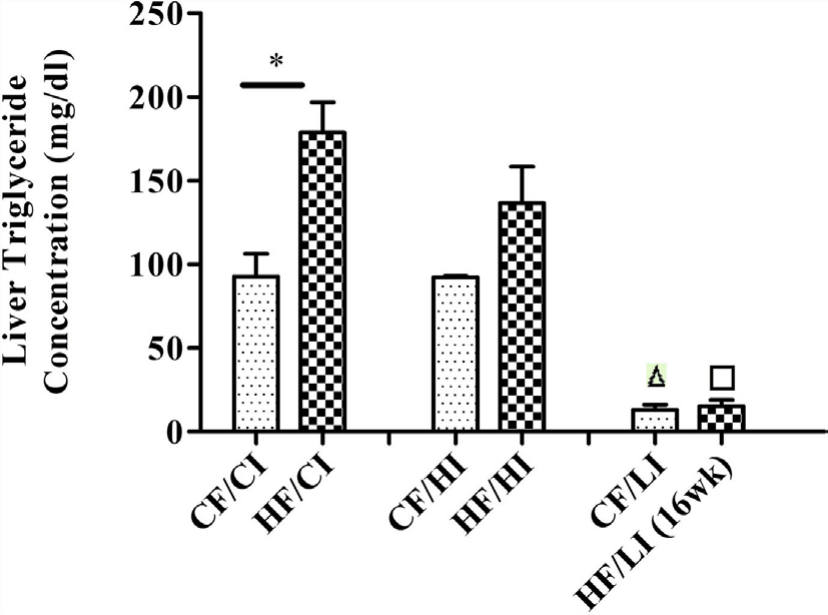
**Mice average liver triglyceride (TG) concentration per treatment group from the initial week to terminal week**. * represents a significant difference in liver TG contents between the control and high fat-treated groups (independent *t*-test, *P* < 0.05). Δ represents a significant difference between the low iron and the control iron-treated groups at the control fat level (independent *t*-test, *P* < 0.05). □ represents a significant difference between the low iron and the control iron-treated groups at the high-fat level (independent *t*-test, *P* < 0.05). Abbreviations: CF, control fat; HF, high fat; CI, control iron; HI, high iron; LI, low iron.

Liver TG concentration was measured to evaluate the effect of high-fat diet (Figure 3). High-fat diet significantly increased the liver TG concentration at the control iron diet level (CF/CI vs HF/CI, *P* < 0.05). There was an interaction between dietary iron and fat on TG content (*P* < 0.05). Low iron diets significantly decreased TG concentrations at both control and high fat diet levels (CF/CI vs. CF/LI, HF/CI vs. HF/LI, *P* < 0.05).

**Figure 4:**
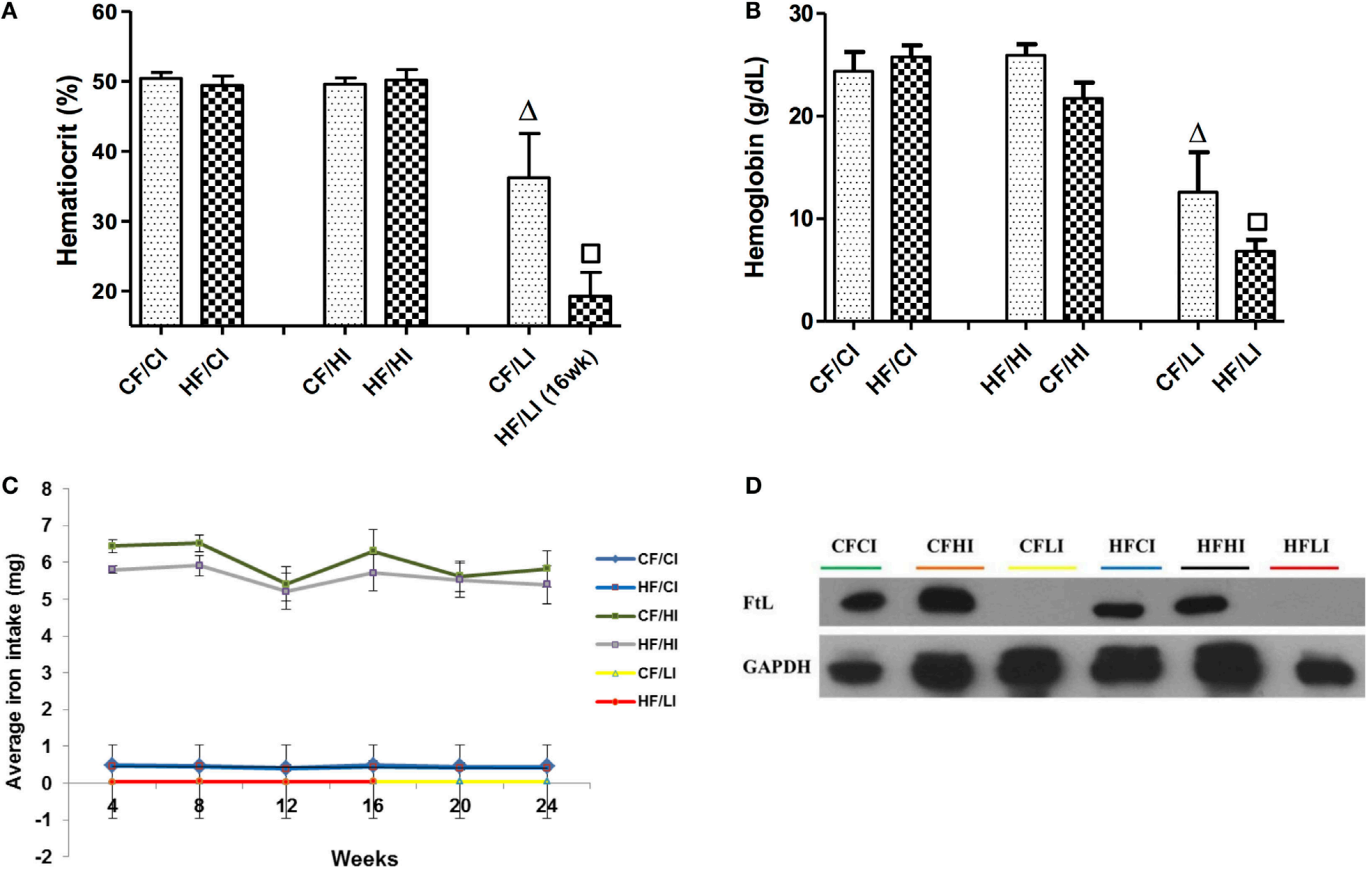
**Hematocrits (A), hemoglobin (B), dietary iron intake (C), and liver ferritin L (D) levels in male C57BL/6J mice in terminal weeks of dietary treatments**. * represents a significant difference between the control and high fat-treated groups (independent *t*-test, *P* < 0.05). Δ represents a significant difference between the low iron and control iron-treated groups at the control fat level (independent *t*-test, *P* < 0.05). □ represents a significant difference between the low iron and control iron-treated groups at high-fat level (independent *t*-test, *P* < 0.05). Abbreviations: CF, control fat; HF, high fat; CI, control iron; HI, high iron; LI, low iron; FtL, ferritin L.

The impacts of dietary iron were measured through hemoglobin, hematocrit, dietary iron intake, and liver FtL expression (Figure 4). Dietary iron had a significant effect on hematocrit and hemoglobin contents (Figures 4A,B). At both control and high-fat diet levels, mice fed with low iron diets exhibited a significantly lower hematocrit and hemoglobin contents compared with control iron groups (CF/CI vs. CF/LI, HF/CI, vs. HF/LI, *P* < 0.05, Figures 4A,B). The average iron intake per mouse per week wash shown in Figure 4C. At both fat levels, the high iron intake was approximately 10 times higher than that in mice fed with the control iron diet, and the low iron intake was about 10 times lower than that in mice fed with the control iron diet (*P* < 0.05). The impact of dietary iron was further confirmed by liver FtL expression in Figure 4D, which showed that low iron diets significantly decreased the FtL expression at both control and high-fat levels.

### Brain Iron Content

**Figure 5:**
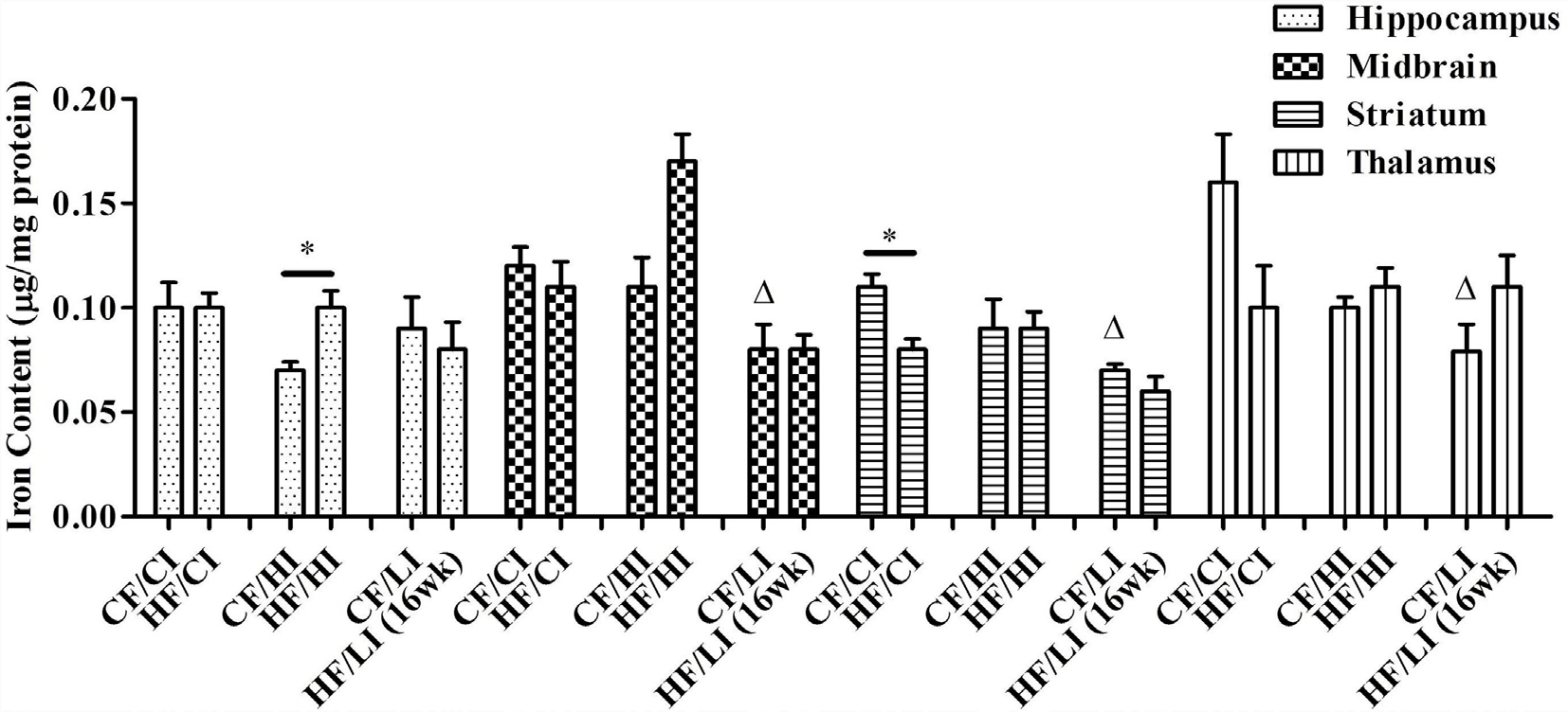
**Brain iron contents in male C57BL/6J mice at the terminal weeks of dietary treatments**. * represents a significant difference between control and high fat-treated groups (independent *t*-test, *P* < 0.05). Δ represents a significant difference between the high or low iron and control iron-treated groups at the control fat level (independent *t*-test, *P* < 0.05). □ represents a significant difference between the high or low iron and control iron-treated groups at the high-fat level (independent *t*-test, *P* < 0.05). Abbreviations: CF, control fat; HF, high fat; CI, control iron; HI, high iron; LI, low iron.

The effects of dietary fat and iron on brain regional iron content were shown in Figure 5. Both dietary fat and iron had significant effects on brain iron contents, but in a regionally specific manner. For example, the striatal iron content was decreased by the high-fat and low iron diets, respectively, and there was no interaction between dietary iron and fat in this region (HF/CI vs. CF/CI, *P* < 0.05, Figure 5). An interaction of dietary fat and iron was found in the hippocampus, midbrain, and thalamus (*P* < 0.05). The HF/HI diet significantly increased brain iron content in the hippocampus compared with control fat fed mice (CF/HI vs. HF/HI, *P* < 0.05). The CF/LI diet significantly decreased the brain iron contents in the midbrain, striatum, and thalamus compared with its control (CF/LI vs. CF/CI, *P* < 0.05).

### Brain Protein and mRNA Expression of Ferritin-H

**Figure 6:**
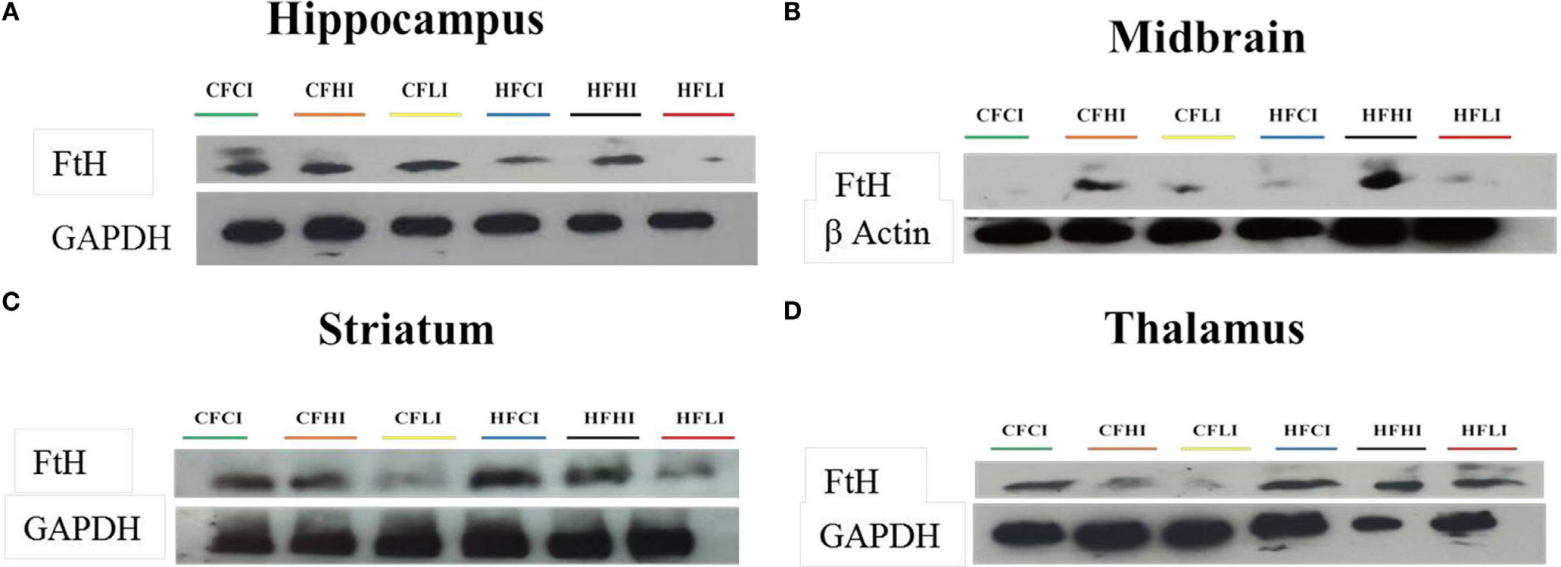
**Ferritin-H (FtH) protein expression in hippocampus (A), midbrain (B), thalamus (C), and striatum (D) tested by Western Blot**.

**Figure 7:**
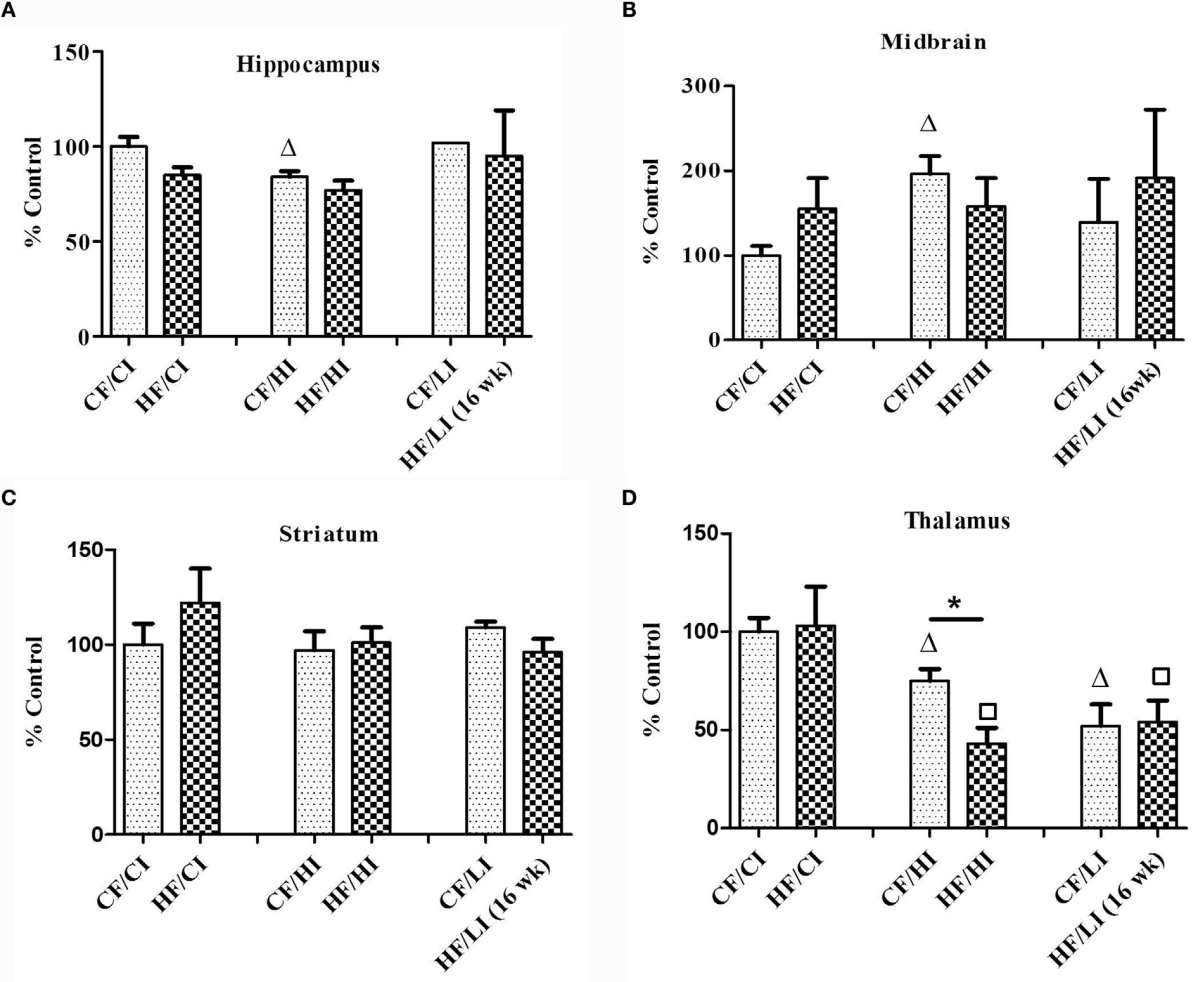
**The mRNA expression of FtH in hippocampus (A), midbrain (B), thalamus (C), and striatum (D) tested by real-time PCR**. Data represented are mean ± SD (*n* = 5). * represents a significant difference between control and high fat-treated groups (independent *t*-test, *P* < 0.05). Δ represents a significant difference between the high or low iron and control iron-treated groups at the control fat level (independent *t*-test, *P* < 0.05). □ represents a significant difference between the high or low iron and control iron-treated groups at the high-fat level (independent *t*-test, *P* < 0.05). Abbreviations: CF, control fat; HF, high fat; CI, control iron; HI, high iron; LI, low iron.

Ferritin heavy chain is an iron-storage protein functioning as an indicator of the cellular iron status. The protein and mRNA expressions of FtH in the hippocampus, midbrain, striatum, and thalamus were studied (Figures 6 and 7). In Figure 6, low iron diet decreased the FtH protein expression at either control or high-fat level in all regions studied. In Figure 7, no dietary interaction between fat and iron was found on the FtH mRNA expression in all regions studied. Dietary fat did not alter FtHmRNA expression level. Only the dietary iron status significantly decreased the FtH mRNA expression in thalamus.

### Behavior Analysis

**Figure 8:**
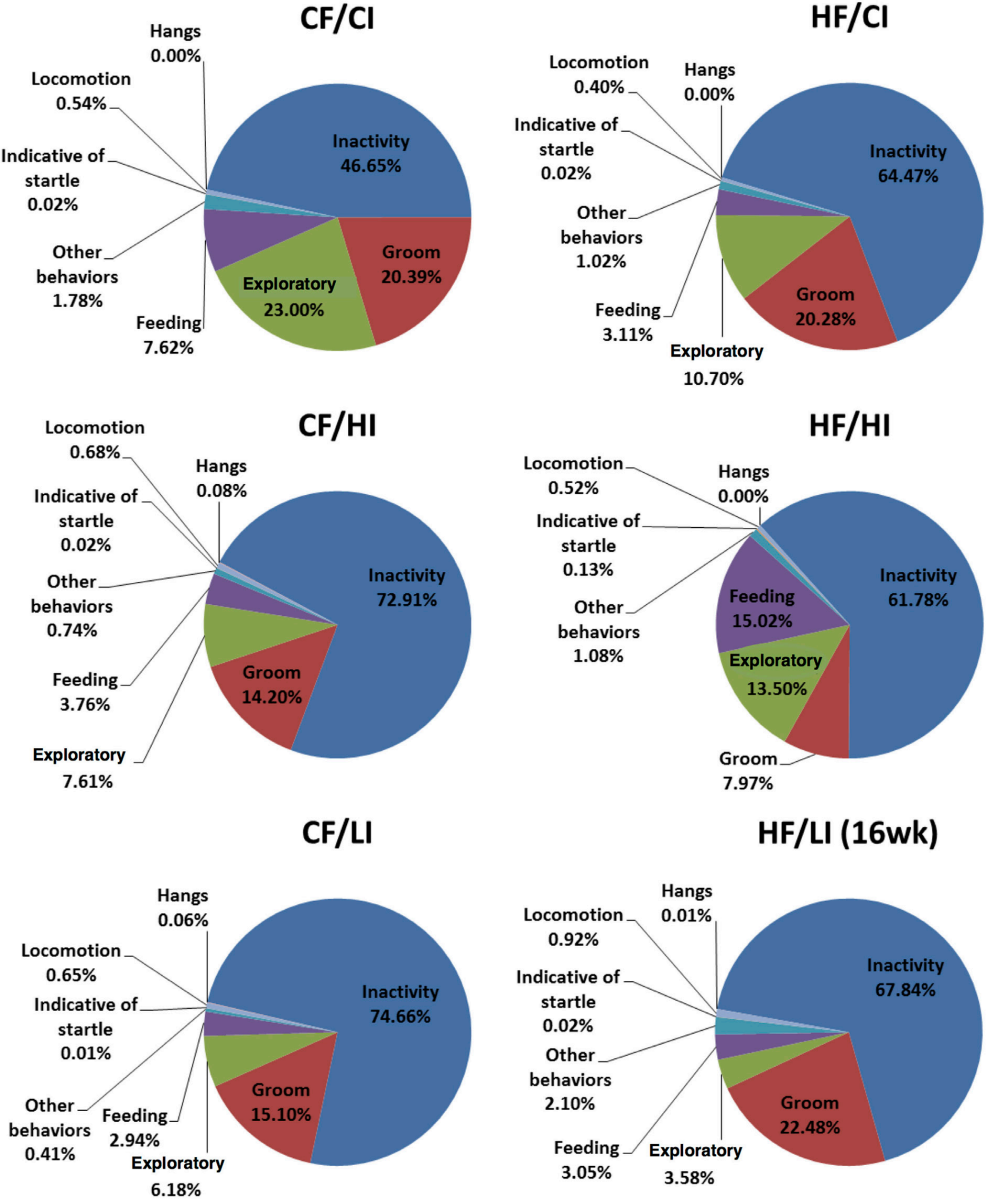
**Behavior patterns of male C57BL/6J mice at the terminal weeks of dietary treatment**. The 24th week was the terminal week for all groups of dietary treatments, except for HF/CI which had the 16th week as the terminal week. Abbreviations: CF, control fat; HF, high fat; CI, control iron; HI, high iron; LI, low iron.

**Figure 9:**
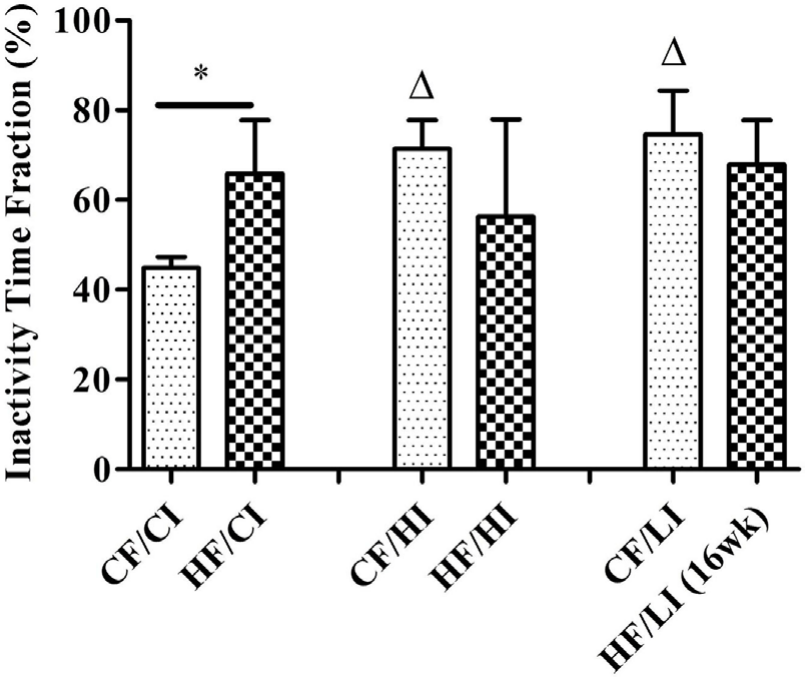
**Inactivity time fractions of male C57BL/6J mice within 24 h at the terminal weeks of dietary treatments**. * represents a significant difference between control and high fat-treated groups (independent *t*-test, *P* < 0.05). Δ represents a significant difference between the high or low iron and control iron-treated groups at the control fat level (independent *t*-test, *P* < 0.05). Abbreviations: CF, control fat; HF, high fat; CI, control iron; HI, high iron; LI, low iron.

**Figure 10:**
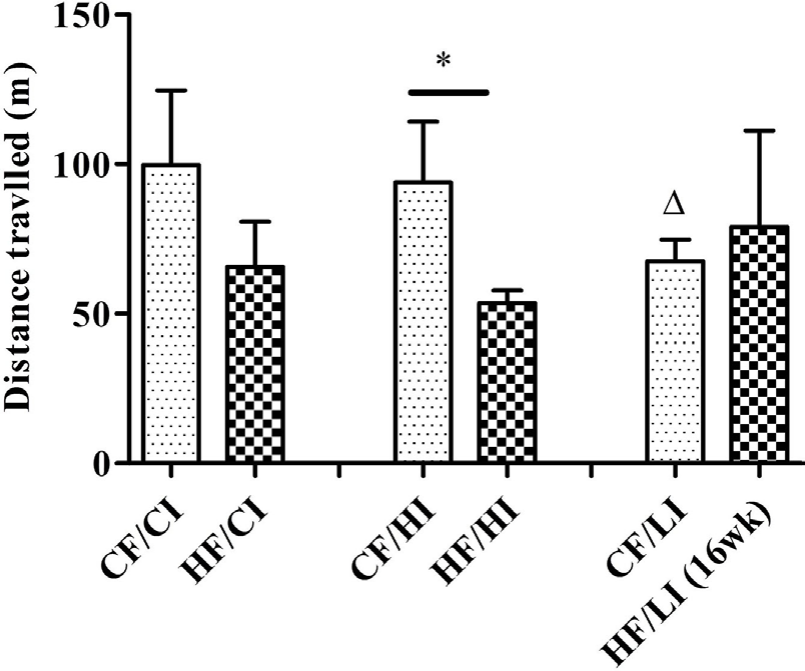
**Total distance traveled (TDT) time fraction of male C57BL/6J mice within 24 h at the terminal weeks of dietary treatments**. * represents a significant difference between control and high fat-treated groups (independent *t*-test, *P* < 0.05). Δ represents a significant difference between the low iron and control iron groups at the control fat level (independent *t*-test, *P* < 0.05). Abbreviations: CF, control fat; HF, high fat; CI, control iron; HI, high iron; LI, low iron.

The overall mouse behavior profile was shown in Figure 8. The stereotypical behaviors, such as inactivity, grooming, locomotion, and feeding, were included in the profile. Inactivity took the largest portion of the profile in all treatment groups. There was an interaction between fat and iron on inactivity (Figure 9, *P* < 0.05). The high-fat diet significantly increased the percentage of inactivity at the control iron level (CF/CI vs. HF/CI, independent *t*-test, *P* < 0.05). Both high and low iron diet increased the percentage of inactivity at control fat level (CF/CI vs. CF/HI, CF/CI vs. CF/LI, *P* < 0.05). Total distance traveled was analyzed in accordance with the results of inactivity. There was also an interaction between iron and fat on the total distance traveled (Figure 10, *P* < 0.05). High-fat diet decreased the total distance traveled at high iron level (CF/HI vs. HF/HI, *P* < 0.05). Low iron diet decreased the total distance traveled at the control fat level (CF/CI vs. CF/LI, *P* < 0.05).

**Figure 11:**
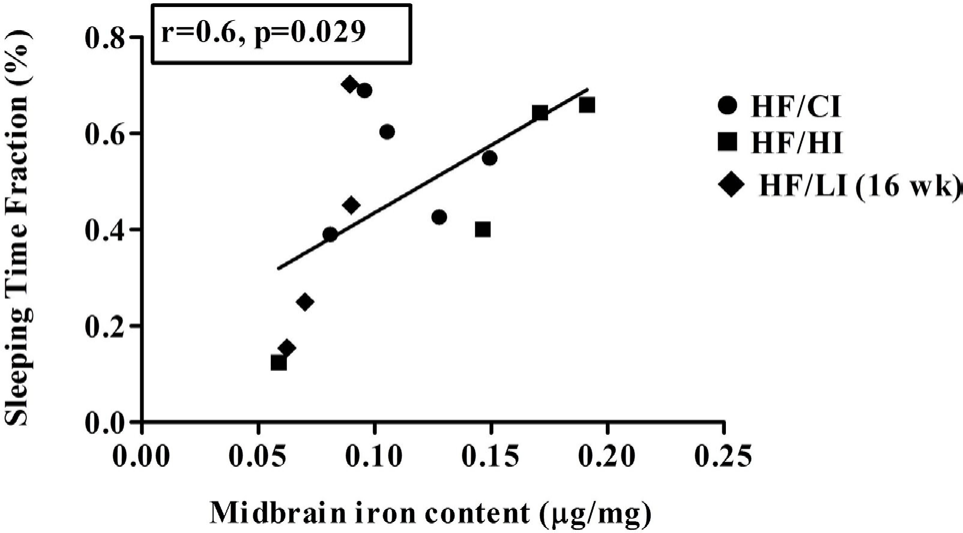
**Positive correlation between midbrain iron content and sleeping at high-fat diet level (*r* = 0.6, *P* < 0.05)**.

The correlations between brain iron contents and the stereotypical behaviors were examined in all regions studied. A positive correlation between midbrain iron content and the sleeping time fraction was found at the high-fat diet level (*r* = −0.6, *P* < 0.05, Figure 11). No other significant correlations were found between brain regional iron contents and stereotypical behaviors.

## Discussion

This is a preliminary study investigating obesity-induced brain regional iron changes. The results showed a heterogeneous iron distribution and regional-specific response to dietary-induced obesity. For example, high-fat diet significantly decreased the brain iron content in the striatum (*P* < 0.05), but not other regions. The high-fat diet altered the brain iron contents and FtH protein and mRNA expressions in a regional-specific manner. Thalamus has a more distinct change in FtH mRNA content compared with other regions. An interaction between dietary iron and fat was found to have a significant impact on brain iron content and stereotypical behaviors. The high-fat diet altered the stereotypical behaviors such as inactivity, locomotion, and total distance traveled (*P* < 0.05). The high-fat diet also led a significant correlation between iron content and sleeping in the midbrain (*P* < 0.05). All these findings contribute toward the research blank of obesity-induced brain iron metabolism.

Only dietary fat decreased the brain iron content in the striatum, but not other regions. The result indicates that only striatum endures a regional-specific ID caused by high-fat diet. The reduction of the striatal iron content has a proven association with the reduction in dopamine D2 receptors (45–47). It has also been reported that brain iron is especially important to dopaminergic modulatory systems, and its deficit would explain the behavioral disturbances such as movement modulation and balances motivation (32, 33, 47). However, due to limited samples, we could not continue analyzing the dopamine expression in striatum to test our hypothesis that striatal iron is associated with dopamine expression. In future investigations, we intend to measure dopamine and its metabolites in the striatum to test the outcome of dietary fat-induced brain iron alteration.

In this preliminary study, we found that high-fat diet altered some stereotypical behaviors and our findings are consistent with the literature. As we expected, the high-fat diet increased the daily percentage of inactivity time fraction at the 24th week of dietary treatments at the control iron level (Figure 9). This result is consistent with other observations that obese subjects are characterized by sedentary behavior (48, 49). The finding that high-fat diets affected the TDT (Figure 10) is also supported by the reports of increased immobility and decreased locomotion activity in both diet-induced and genetically obese mice (50, 51).

Since evidence showed that behavioral alteration is tightly associated with metabolic defects (52), we hypothesized that the alteration of the behavior is due to the changes in brain iron content caused by high-fat diet. However, our data did not support this hypothesis. The correlations between brain iron contents and stereotypical behaviors were tested in all regions. Only one positive correlation between sleep time fraction and midbrain iron contents was found at the high-fat diet level (Figure 11, *P* < 0.05). Since the correlation does not indicate a causation, it is inconclusive if the correlation between midbrain iron and sleeping time is due to the regional iron change.

This study included various levels of iron and fat diets to examine not only the effects of dietary fat and iron, respectively, but also their interactions on brain iron biology. The purpose of adding high or low iron groups was to illustrate the consequence of the interaction between dietary iron and fat. We found that mice fed with HF/LI diet experienced ulcerative dermatitis, which is a different disease than severe ID. To be ethical to HF/LI fed mice, as soon as we found the disease symptoms, we removed this group of mice at week 16. The data from HF/LI mice should not be included in the figures in comparison with mice terminated at 24th week. However, we think that it will be very informative to see the outcome of HF/LI in comparison to control mice. Regarding the high iron diet, it was due to our expectation that high-fat diet might decrease brain regional iron contents the same as it does to the systematic tissues. We assumed that high iron diet might minimize the possible ID caused by high-fat diet; however, our data showed that the effect of interaction between high iron and high-fat play a distinct role rather than high iron itself. The future study could investigate the effect of iron repletion after the symptoms of ID induced by high-fat diet is observed.

The major limitation of the study was the small number of animals. With only five mice per group, the conclusion made from this preliminary study should be confirmed with a larger animal size. The strength of this paper is that it is the first study to explore brain iron changes and its relation to behavior under the effect of the high-fat diet. The conclusion that dietary fat and iron resulted in the heterogeneous iron distribution in brain regions will lay foundations to further explore the disease processes of obesity where altered iron may be implicated.

## Author Contributions

LL and AB carried out the physiological and behavior experiments. JP measured brain iron contents and ferritin mRNA expressions. JH and KE designed the study. All authors participated in the data analysis. LL, KE, SH, and JH wrote the manuscript. All authors read and approved the final manuscript.

## Conflict of Interest Statement

The authors declare that the research was conducted in the absence of any commercial or financial relationships that could be construed as a potential conflict of interest.
